# Some Chronic Joint Lesions

**Published:** 1909-01-16

**Authors:** 


### Surgery.

### SOME CHRONIC JOINT LESIONS.

There are few surgical conditions which are more troublesome to treat and give less satisfactory results than chronic inflammation in a joint following an injury; and the two joints, of which this is particularly true, are the shoulder and the knee. This is not surprising: the knee is a joint which is adapted for standing in the upright position and for walking. For this reason the articular surfaces are very large, and the ligaments connecting them are of great strength; and in a normal knee the only movements allowed are those of flexion and extension. But it is not well protected from injury, because the ends of the bones in this situation lie very superficially; and then, again, although the ligaments are strong, they have little or no power of resisting any lateral or spiral force, and it is in this way that injuries of the knee-joint are produced, commonly by a side-slip when walking or by football. This game is a fertile source of joint injuries, and particularly of injuries of this joint.

The history in these cases is always of somewhat the same type. In a short time after the accident acute simple synovitis ensues. The joint is filled with fluid, and is then held in the position of greatest comfort, or rather, one should say, of least discomfort. This is a position of slight flexion, since thus the synovial membrane can accommodate the greatest quantity of free fluid. Walking is very painful, so that the patient limps noticeably. The question as to whether there is free fluid in a knee-joint or not is one that is easily settled by simply looking at the joint. The normal hollows of the articulation will be seen to have disappeared and to have been replaced by swellings. In a typical case a semilunar swelling can be seen above the upper border of the patella: this is due to an accumulation of fluid in the subcrural pouch; and again a small swelling can be seen on either side of the ligamentum patellæ. These changes are less obvious in a woman than in a man, because normally in a woman's knee the hollows are less well marked. But in any case the appearances described are characteristic of free fluid, and more reliance may be put on them than on the presence of "the patellar tap," which is not so valuable a sign. The latter depends on the fact that when there is fluid on the joint the patella floats upon it, and if depressed with the finger, can be felt to come in contact with the condyles of the femur.

The treatment of the case in the acute stage resolves itself into rest and the adoption of measures to promote the absorption of the fluid. It is best to put the patient to bed with the limb on a McIntyre's splint, at such an angle that the flexed limb lies in it comfortably, and to apply an anodyne lotion—*e.g.* lotio plumbi cum opio. The splint can be straightened by slow degrees daily, until in a few days the limb can be kept quite straight. The acute pain will usually also have disappeared by then. As soon as this has occurred, active means may be taken to hasten the absorption of the fluid. In the writer's opinion the best treatment is to apply a crêpe velpeau bandage firmly round the joint. This is better than an elastic knee-cap or a Martin's elastic bandage, both of which are hot and irritating. At the same time the patient may be encouraged to use the limb, since even slight movement is sufficient to open up the lymphatic stomata in the synovial membrane, and this assists the absorption of the fluid; conversely, while the leg is absolutely at rest this does not occur so readily. As soon as the fluid has subsided and the joint returned to its normal size and appearance, massage may be begun with great advantage, because it strengthens the periarticular ligaments and tendons which have been weakened by the distension of the joint in the acute stage.

So far all is comparatively simple, but it is later that the joint causes the patient so much trouble. However well, therefore, the acute stage has reacted to treatment, it is wise not to give too good a prognosis. It must be remembered that all the structures in the joint have been stretched by the tension to which they have been subjected, and they are never quite as efficient as they were before. The resulting condition is one which has been well called "the wobbly knee." It is not pain that the patient complains of so much as a sensation of weakness in the joint, which is brought on by exercise, or more particularly by prolonged standing on it. There may be recurrent attacks of slight effusion, which render extreme flexion or extension painful. In other cases the weakness becomes so great that the patient suddenly falls to the ground when walking, and the condition may be mistaken for one of displaced semilunar cartilage. But in an ordinary case of chronic synovitis the joint is not locked in a position of flexion as it is in that condition.

The writer has a more than ordinarily intimate knowledge of this condition, because he is himself a sufferer from it. While at school he fell from a horizontal bar in a gymnasium with the right knee acutely flexed under him. Acute synovitis followed, and the effusion did not go down for four or five weeks. After this the knee was always weak, but nothing caused such discomfort as the necessity of standing still in the operating theatre while fulfilling the duties of a dresser, on some occasions for several hours on end.

There is no treatment which can be regarded as holding out any prospect of a permanent cure for this condition. When an attack of subacute synovitis comes on, as the result of prolonged standing or exercise, it is best treated by rest and the application of the crêpe bandage, as in the subsiding stages of the acute synovitis. But more than this, the bandage should be worn for some time after the effusion has subsided, because it supports the articulation and, in part, at any rate, dispels the great feeling of weakness which is the outstanding feature that troubles the patient.

The ordinary forms of counter irritation such as Scott's dressing are of little value. In very obstinate cases washing out the joint has been advised, but this is an extreme measure and one not to be undertaken lightly.

